# Potential of the Dietary Antioxidants Resveratrol and Curcumin in Prevention and Treatment of Hematologic Malignancies

**DOI:** 10.3390/molecules15107035

**Published:** 2010-10-12

**Authors:** Mareike Kelkel, Claus Jacob, Mario Dicato, Marc Diederich

**Affiliations:** 1 Laboratoire de Biologie Moléculaire et Cellulaire du Cancer, Fondation de Recherche Cancer et Sang, Hôpital Kirchberg, 9 Rue Edward Steichen, 2540 Luxembourg, Luxembourg; E-Mail: mareike.kelkel@lbmcc.lu (M.K.); 2 Division of Bioorganic Chemistry, School of Pharmacy, Saarland University, 66123 Saarbruecken, Germany; E-Mail: c.jacob@mx.uni-saarland.de

**Keywords:** dietary polyphenolic antioxidants, resveratrol, curcumin and derivatives, leukemia

## Abstract

Despite considerable improvements in the tolerance and efficacy of novel chemotherapeutic agents, the mortality of hematological malignancies is still high due to therapy relapse, which is associated with bad prognosis. Dietary polyphenolic compounds are of growing interest as an alternative approach, especially in cancer treatment, as they have been proven to be safe and display strong antioxidant properties. Here, we provide evidence that both resveratrol and curcumin possess huge potential for application as both chemopreventive agents and anticancer drugs and might represent promising candidates for future treatment of leukemia. Both polyphenols are currently being tested in clinical trials. We describe the underlying mechanisms, but also focus on possible limitations and how they might be overcome in future clinical use – either by chemically synthesized derivatives or special formulations that improve bioavailability and pharmacokinetics.

## 1. Introduction

With more than 3 million new cases and 1.7 million deaths each year, cancer is the most important cause of death and morbidity in Europe after cardiovascular diseases. According to the WHO, it accounts for 20% of all deaths in Europe, a rate which is even believed to increase in the future. The most recent cancer statistics resource, GLOBOCAN 2008, counted 47,500 new cases of leukemia and more than 32,000 deaths in European men. Thereby, leukemia reached seventh place on the list of the ten most frequent cancer types in men. In women, leukemia is less frequent, currently being in place 11. Leukemia develops from hematopoietic stem cells that escape the normal control mechanisms thereby interrupting their capacity to differentiate in mature blood cells [1,2,3]. As a result of uncontrolled proliferation of hematological progenitor cells, excessive number of malignant cells accumulates in the bone marrow where they replace normal marrow tissue and affect physiological production of blood cells. 

In this review we will focus on the role of oxidative stress in leukemogenesis and how the natural antioxidants resveratrol and curcumin interfere with and prevent this process. We provide evidence for their impressive chemopreventive and chemotherapeutic potential by delivering insight in the detailed action of both compounds, their additional cellular targets beside free radicals and the signaling pathways affected. We also highlight the role of their pro-oxidant effects and present an overview about the efforts that were undertaken in order to improve bioavailability and pharmacokinetics of resveratrol and curcumin.

### 1.1. Role of Oxidative Stress and Cellular Antioxidant Defense Mechanisms

Reactive oxygen species (ROS) including superoxide (O_2_^•−^), hydroxyl (OH^•^) and peroxyl radicals (ROO^•^) or hydrogen peroxide (H_2_O_2_), even if they have some physiological functions, exert deleterious effects when present at high concentrations. They cause oxidative damage on cellular DNA, proteins and lipids [4]. Beside ROS, reactive nitrogen species (RNS), including nitric oxide (NO^•^), play a role in oxidative damage of proteins *via* nitrosylation reactions. More importantly, NO^•^ can act in combination with superoxide to produce a highly reactive peroxynitrite anion (ONOO^−^), which subsequently triggers DNA fragmentation and lipid peroxidation [5]. Such permanent modifications of cellular macromolecules might ultimately result in carcinogenesis [6,7,8,9,10,11,12].

**Figure 1:**
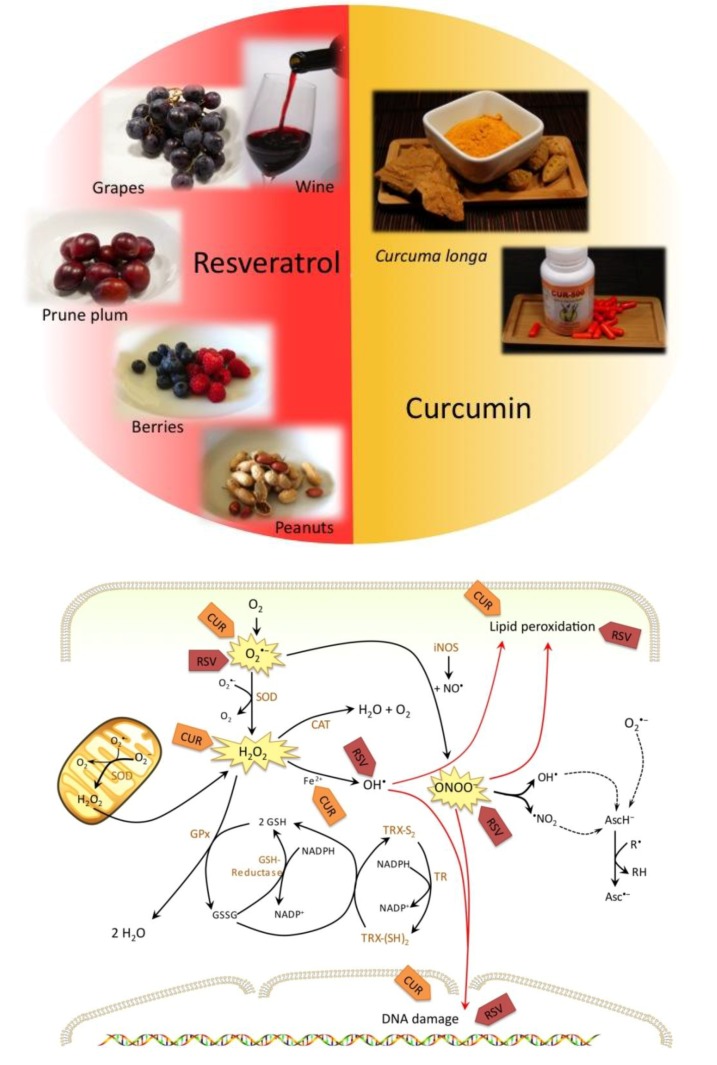
**(a)** Resveratrol and curcumin are two intensively studied polyphenols. **(b)** Molecular targets of resveratrol (RSV) and curcumin (CUR).

In order to prevent harmful accumulation of damaged DNA, lipids and proteins and subsequent initiation of carcinogenesis the cell possesses a complex and highly effective system of antioxidant defense that allows an immediate response to oxidative stress. Various enzymatic antioxidants like superoxide dismutase (SOD), catalase (CAT) and glutathione peroxidase (GPx) as well as non-enzymatic antioxidants act together to render ROS/RNS and H_2_O_2_ harmless. Cytosolic or mitochondrial forms of SOD catalyze the conversion of superoxide anion (O_2_^•−^) to H_2_O_2_ and O_2_. The resulting H_2_O_2_ is subsequently removed by the enzymatic activity of GPx or CAT. The latter is localized in peroxisomes and converts H_2_O_2_ with an impressive turnover rate to water and molecular oxygen [13]. GPx on the other hand acts in both cytosol and mitochondria where it counteracts oxidative stress by reducing peroxides to water with simultaneous oxidation of glutathione (GSH) to glutathione disulfide (GSSG) [14]. GSH is a non-enzymatic antioxidant present in cytosol, nucleus and mitochondria that consists of three amino acids and represents the major thiol-disulfide redox buffer, responsible for maintenance of the overall redox balance in the cell [15]. It plays an important role in the regulation of redox sensitive cysteine-containing enzymes [16,17] and serves as cofactor for detoxifying enzymes like GPx, which in turn prevent lipid oxidative damage by reducing lipid peroxides [18]. Moreover, GSH serves as direct ROS scavenger and functions in the regeneration of oxidized forms of antioxidant vitamins C and E [13]. Due to the fact that GSH modulates activation and binding of transcription factors [17] and as the cellular concentration of the reduced GSH is up to 100 fold higher than GSSG, minor increase in GSH oxidation can significantly affect the GSH:GSSG ratio and consequently influences signal transduction and cell cycle progression [19]. The balance can be restored by NADPH-dependent glutathione reductase or the thioredoxin/glutaredoxin systems that catalyze the inverse reaction by reducing GSSG to GSH or by elimination of the oxidized GSSG from the cell [20]. Beside glutathione, the thioredoxin system significantly contributes to the intracellular redox environment. Two cysteine residues within the active site of thioredoxin (TRX) are responsible for its ability to reduce disulfide bonds within GSH or multiple oxidised proteins, including several transcription factors [21]. Thioredoxin reductase (TR) afterwards catalyzes the NADPH-dependent reduction of oxidized TRX into its active form. Another essential part of the non-enzymatic antioxidant defense is the ascorbate system. Vitamin C (ascorbic acid), a diacid with two ionizable hydroxyl groups, exists at physiologic pH mainly in its AscH^−^ form. Interaction with ROS leads to ascorbate-derived products that are less reactive and the resulting Asc^•−^ radical represents the terminal small-molecule antioxidant [13]. Vitamin C is a hydrophilic antioxidant that acts together with the membrane-localized vitamin E in protecting membrane lipids from peroxidation as it regenerates oxidized vitamin E [22]. Figure 1b gives an overview about the cellular antioxidant defense and simultaneously indicates molecular targets of resveratrol and curcumin.

### 1.2. Role of Oxidative Stress in Development and Evolution of Leukemia

Over the last years evidence has accumulated that oxidative stress might be an important player in hematological malignancies. In patients suffering from different kinds of leukemia the balance between free radicals (ROS/RNS) and cellular antioxidant defense mechanisms is disturbed. A study performed on 20 patients with chronic leukemia indicated that leukemic cells from these patients produce more ROS than non-leukemic cells. Moreover, the total antioxidant activity in these cells was not sufficient to antagonize the harmful effects of free radicals [23]. In lymphocytes of chronic lymphocytic leukemia (CLL) patients for example, Oltra *et al.* [24] measured lower SOD and CAT activities, progressively decreasing within four years of CLL disease without chemotherapeutic treatment. Since oxidative stress causes stable chromosome modifications and mutagenesis represents an important trigger of cancer development, such redox imbalance is tightly associated with oncogenic stimulation. Mutagenesis is induced in response to a moderate level of oxidative stress. Weak oxidative conditions on the other hand play a role in tumor promotion, whereas high levels of free radicals are involved in apoptosis [25]. Accordingly, oxidative damage on DNA and lipids was accumulating in CLL patients over time, shown by raised levels of the oxidation products 8-oxo-dG and malondialdehyde (MDA) [24]. These results are in agreement with earlier findings that revealed higher levels of DNA base lesions and lower levels of the antioxidant enzymes SOD and CAT in patients with childhood acute lymphoblastic leukemia (ALL) compared to levels in healthy children [26]. Oxidative DNA damage afterwards might influence transcriptional regulation and introduce replication errors, cause modulation of signaling pathways or lead to genomic instability thereby promoting cancer development [27]. GPx activity was also reduced in lymphocytes of ALL patients compared to control lymphocytes [26]. This observation seems to be specific for acute forms of leukemia as GPx activity was actually elevated in CLL [24,28], possibly as part of an adaption mechanism against hydrogen peroxide. A clinical study from Zhou *et al.* indicated that there is also a strong correlation between oxidative stress and the development of acute myeloid leukemia (AML) as well as the incidence of disease relapse [29]. In 102 leukemic patients in the primary condition or at relapse, oxidative stress levels were significantly increased compared to 102 healthy volunteers tested. Many AML patients successfully treated with chemotherapeutics undergo a subsequent relapse that is responsible for poor survival rates. The mean plasma level of the lipid peroxidation product and most popular marker for oxidative stress, MDA, was found significantly higher at relapse stage whereas the total antioxidant capacity was decreased to half its initial level [29]. These findings allow the conclusion that oxidative stress persists in relapsing AML patients. A further study on pediatric patients with ALL provided evidence that increases in oxidative damage on proteins and lipids in these patients, measured by protein carbonylation and MDA level respectively, was not caused by chemotherapy. Their results indicated that the accumulation of oxidative lesions in non-treated patients and patients in the first phase of treatment was higher than in those treated for longer times or out-of-treatment. Antioxidant activities of CAT and SOD on the other hand were simultaneously decreased [30]. In a previous study, Zhou *et al.* tested 92 AML patients, from which 48% were suffering from clinical depression. Compared to the control group, in depressive patients significantly elevated serum concentrations of ROS, NO and MDA could be detected. This increase of free radicals and oxidative damage was accompanied with decreased total antioxidant capacity and SOD levels [31]. 

All these observations argue for a link between decreased levels of the cellular antioxidant defense and accumulation of oxidative damage in this type of cancer. As the biggest differences in the activity of antioxidant enzymes have been detected in early stages of the disease, it is supposable that persistent oxidative stress plays a role in the development of leukemia [30]. This theory is corroborated by results of an *in vivo* study performed on high leukemic mice. During the development of natural lymphatic leukemia in these mice, the activities of SOD, GPx and CAT decreased significantly [32]. Moreover, persistent oxidative stress could be observed in depressive and relapsing patients of AML, indicating its importance also at these stages [29,31].

It is worth to mention that oxidative stress is associated with inflammation. A few years ago, a connection between the long-term intake of anti-inflammatory drugs, such as aspirin as well as some natural compounds, and a reduced incidence of cancer has been demonstrated [33,34,35]. These data confirm results from epidemiological and experimental studies indicating that chronic inflammation is linked to carcinogenesis [36,37]. An inflammatory stimulus causes oxidative stress *via* accumulation of ROS and induces lipid peroxidation as well as miscoding DNA adducts, which are then directly implicated in the initiation of carcinogenesis [38,39]. Simultaneously, ROS leads to the modulation of redox-sensitive transcription factors and thereby activates NF-κB- and other signaling pathways. As a consequence, aberrant expression of their target genes creates a dangerous feedback loop. Pro-inflammatory cytokines and chemokines for example are known to play a role in tumorigenesis [40,41]. They lead not only to an increased production of ROS in phagocytic and non-phagocytic cells but also recruit further inflammatory cells [39,42].

### 1.3. Natural Antioxidants in the Treatment of Hematologic Malignancies

As the level of the total cellular antioxidant capacity, including antioxidant enzymes as well as non-enzymatic antioxidants, is decreased during the etiology of leukemia as explained above, administration of antioxidants might represent a successful way to overcome hematologic malignancies. In nature, manifold antioxidants are produced in dietary plants and many of them are already evaluated *in vitro* or *in vivo* as potent anti-carcinogenic agents in different kinds of tumors. They are tested alone or in combination with other antioxidants or classical chemotherapeutics and some of them even revealed promising results in clinical trials. As classical chemotherapy is often associated with severe side effects and hardly affordable due to its high costs, researchers are focusing their attention now on the search for alternative medicines. In this respect, natural compounds are again of growing interest and became intensively investigated within the last years. For many plant derived compounds, epidemiological studies indicated that there is a correlation between the dietary intake and reduced incidence of inflammation and cancer. Interestingly, some of them are used since centuries in traditional medicine all over the world. Compared to classical treatment, they possess several important advantages. Beside lower costs and good availability of natural compounds, it is noteworthy that they do not exert serious side effects on normal tissues and most strikingly, these compounds can, in contrast to classical chemotherapeutics, even be used for chemoprevention.

In this review we present the two most promising naturally derived antioxidants, which belong to the group of polyphenols and have been intensively studied over the last years [Figure 1(a) and Figure 1(b)]: resveratrol and curcumin. We deliver detailed insight in their antioxidant but also pro-oxidant effects and provide evidence for the potent antileukemic action of these compounds and some derivatives.

## 2. Resveratrol

The non-flavonoid polyphenolic compound resveratrol (1) is well known from the so-called “French paradox”, describing the epidemiologic observation of an inverse correlation between red wine consumption and the incidence of cardiovascular disease [43]. Since 1976 it is known that resveratrol is a phytoalexin produced in high amounts in the skin of grape berries in response to infection or mechanical injury [44], subsequently accounting for concentrations up to 7.7 mg/L resveratrol in red wine [45]. Besides in grapes this *trans*-3,5,4'-trihydroxystilbene is abundant in more than 70 plant species including berries, peanut and several herbs [46,47] and confers many health beneficial activities, acting also against inflammation and carcinogenesis [48,49,50]. It is well known that carcinogenesis is a multi-step process subdivided in three stages: tumor initiation, promotion and progression. The chemopreventive potential of resveratrol is based on its adverse effects on processes involved in every single step of tumor development [46,50,51].

### 2.1. Antioxidant Effects of Resveratrol Prevent Initiation of Carcinogenesis

ROS and other free radicals are able to interact with DNA to induce mutations and DNA base modifications. This oxidative damage represents the initial step of carcinogenesis when cellular repair mechanisms fail to fix these lesions [13,52,53] and result in either modulation of gene expression through epigenetic effects or in permanent somatic mutations and chromosomal rearrangements. The antioxidant activity of resveratrol is mainly responsible for inhibition of tumor initiation by preventing free radicals from interacting with cellular DNA. In leukemic HL-60 cells for example resveratrol has been shown to anticipate free radical formation induced by 12-*O*-tetradecanoylphorbol-13-acetate (TPA) [50]. Jang *et al.* provided evidence that resveratrol strongly inhibits ROS production in human monocytes and neutrophils [54]. Recently, Burkitt and Duncan described the powerful antioxidant action of resveratrol in the presence of the ascorbate or glutathione system. Their results revealed that resveratrol protects cells from DNA damage *via* classical hydroxyl-radical scavenging activity in the ascorbate system and by a novel mechanism including the inhibition of glutathione disulfide bond formation [55]. Shamon *et al.* further reported that resveratrol prevents mutagenesis in7,12-dimethylbenz(a)anthracene (DMBA)-treated *Salmonella typhimurium* [56]. An *in vivo* study performed on a mouse skin cancer model indicated that tumorigenesis is significantly reduced in DMBA-treated mice when 1 to 25 μmol resveratrol are administered together with the phorbol ester 12-*O*-tetradecanoylphorbol-13-acetate (TPA) and in addition, no toxicity due to resveratrol could be observed [50]. Another mentionable feature linked to tumor initiation is the ability of resveratrol to induce phase II detoxification enzymes like NAD(P)H: quinone oxidoreductase [57] which has been shown to protect cells from toxicity and neoplasia [58,59]. Some years ago Chen *et al*. found that likewise heme oxygenase-1 (HO-1), another component of the cellular antioxidant defense, is upregulated by resveratrol. Deeper investigation of the underlying mechanism revealed that Akt and ERK1/2 kinases are activated in response to resveratrol treatment and that these signaling pathways finally increase levels of NF-E2-related factor 2 (Nrf2) [60]. This redox-sensitive transcription factor represents a potent activator of anti-oxidant response element (ARE)-depending genes [61] including various phase II detoxifying enzymes. The antioxidant activity of HO-1 is due to catabolism of heme, which is converted into biliverdin. The latter is afterwards metabolized into the antioxidant bilirubin [62].

Evidence was provided that resveratrol specifically inhibited cyclooxygenase and hydroperoxidase activities of cyclooxygenase (COX)-1 and likewise inhibits COX-2 [63,64]. This finding is particularly important as COX can convert carcinogens into DNA-damaging forms [50] and suppression of COX function leads to disruption of prostaglandin biosynthesis [51]. As prostaglandins are important players in pathogenesis of both inflammation and cancer, the latter effect is an important example for the antitumor promotion activity of resveratrol. Beside the possibility of a direct DNA damage caused by ROS, mutations and genotoxicity can also result indirectly from lipid peroxidation. Phospholipids of cellular membranes are extremely susceptible to oxidation due to their high content of polyunsaturated fatty acids, which participate in free radical chain reactions. Initial products, such as lipid hydroperoxides, afterwards produce reactive aldehydes and epoxides in the presence of metals [13]. The most prominent one is MDA, which is not only mutagenic and carcinogenic in mammals [65,66] but further reacts with DNA bases to form deleterious adducts like M1G, M1A or M1C. Other DNA adducts caused by lipid peroxidation are exocyclic etheno adducts [13]. Among these, especially etheno-dA and etheno-dC are of great importance since they act mutagenic on monkey kidney cells [67]. To protect phospholipids from oxidation therefore represents another important strategy to counteract tumor initiation. Like vitamin E, resveratrol is a lipid antioxidant that has the ability to prevent lipid peroxidation by scavenging peroxyl radicals within the membrane [68]. Murcia *et al.* even found that its ability to avoid lipid peroxidation was higher compared to the one associated with vitamin E, just as its HOCl scavenging property [69]. This is in agreement with findings from Stojanovic *et al.* who analyzed reactions of lipid peroxyl radicals with natural antioxidants. They reported that the radical scavenging activity of resveratrol is comparable to that of the flavonoids epicatechin and quercetin, yet exceeds that of vitamins E and C [70]. Moreover, it has been shown that *trans*-resveratrol is able to defend low-density lipoprotein (LDL) from copper-mediated oxidation *via* scavenging free radicals and - more importantly - through its potential to chelate copper [71]. Compared to other polyphenols one advantage of resveratrol worth mentioning is that this compound does not chelate iron, hence it does not affect iron absorption [72]. Taken together, the potent antioxidant activity of resveratrol is mainly responsible for its important cancer chemopreventive effects since free-radical induced lipid peroxidation and oxidative damage of DNA are causative factors in cancer development [73].

### 2.2. Regulation of Cell Cycle, Proliferation and Apoptosis by Resveratrol Affects Cancer Promotion

Beside its ROS scavenging activity, resveratrol has been shown to interact with many cellular targets. It interferes with different signaling pathways and even exhibits some pro-oxidant activities under certain conditions that are linked to antitumor promotion and progression. By using 2',7'-dichlorfluorescein (DCFH) measurements, De Salvia found a slight ROS accumulation in resveratrol treated CHO cells, whereas resveratrol did not induce primary DNA damage. Further results indicated resveratrol-mediated induction of chromosome aberrations in a dose-dependent manner [74]. Gautam *et al.* demonstrated that resveratrol caused apoptotic DNA fragmentation in three leukemia cell lines (32Dp210, L1210, HL-60) but not in normal bone marrow cells [75]. Oxidized products of resveratrol were generated in leukemia cells following the resveratrol-catalyzed reduction of Cu^2+^ to Cu^+^ that increased generation of DNA strand breaks [76]. Long-term administration of resveratrol on HCT-116 cells in sub-apoptotic concentrations resulted in growth arrest caused by a chronically enhanced ROS level and activated the DNA damage checkpoint [77]. These data revealed a new link between the pro-oxidant activity of resveratrol and induction of cell cycle arrest. In fact, several studies demonstrate antiproliferative effects of resveratrol on various leukemic cell lines. For instance, Bernhard *et al.* have shown that resveratrol induces S-phase arrest in T-cell derived acute lymphocytic leukemia cell line CEM-C7H2 followed by Fas-independent apoptosis [78]. S-phase arrest occurs also in AML cells in response to resveratrol treatment by reducing interleukin (IL)-1β expression and subsequent suppression of NF-κB activation [79]. One plausible mechanism by which resveratrol might mediate cell cycle arrest in the S-phase involves inhibition of DNA synthesis. Previous studies reported that resveratrol affects DNA replication through inhibition of ribonucleotide reductase and DNA polymerase activity [46,80]. In detail, it has been shown that the 4'-hydroxy group of *trans*-resveratrol is required for the antiproliferative effect and probably interacts with DNA polymerases α and γ [81,82]. The pro-apoptotic action of resveratrol on T-cell lymphotrophic virus-1-infected cell lines further correlates with suppression of survivin expression [83]. Beyond, *trans*-resveratrol induced cell death in B-CLL cell lines *in vitro* as well as in *ex vivo* models, that was associated with typical apoptotic features like activation of caspase-3, loss of mitochondrial membrane potential and down-regulation of the two antiapoptotic proteins Bcl-2 and inducible nitric oxide synthase (iNOS) [84,85]. INOS is spontaneously expressed in leukemic cells and tumor cells in which the iNOS pathway is blocked are progressively driven into apoptosis. Although the underlying mechanisms responsible for the antiapoptotic role of ^•^NO are not known in detail, they might involve inhibition of caspase activation and loss of mitochondrial membrane potential [86]. 

As mentioned before, resveratrol is also known to affect signaling pathways. Bhardwaj and colleagues for example found both NF-κB and STAT3 pathways inhibited in human multiple myeloma cells treated with resveratrol. Concomitantly, several antiapoptotic proteins like Bcl-2, Bcl-xL, XIAP and survivin were downregulated, consequently sensitizing cells to caspase-dependent apoptosis [87]. By this mode of action resveratrol might be able to overcome chemoresistance, which is closely associated with constitutive activation of these two signaling pathways [87]. These results are in line with observations of Youn *et al*. who found that resveratrol affected activation of NF-κB, STAT3 and ERK and moreover downregulated iNOS expression in mouse colitis [88]. Beside NF-κB, Kundu *et al*. identified another transcription factor, AP-1, as cellular target of resveratrol. The latter inhibited DNA binding of AP-1 and prevented expression of some AP-1 components in the nucleus of mouse skin cells upon TPA stimulation [89,90]. Tili *et al*. found that resveratrol reduced AP-1 activity in human THP-1 monocytic cells and blood monocytes by upregulating non-coding, tumor-suppressor micro-RNA *miR-663*. Simultaneously, this upregulation of *miR-663* leads to a decrease of *miR-155*, which is highly expressed in many human cancers. Upregulation of *miR-663* might therefore represent a strategy to improve the positive effects of resveratrol as anti-cancer agent [91,92]. As a result of disturbing these pro-inflammatory signaling pathways (TPA-induced) expression of the tumor promoter COX-2 was suppressed [89,93]. The inhibition of NF-κB is not restricted to leukemias as this pathway was also impaired in human pancreatic cell lines, in which resveratrol caused apoptosis with inhibition of Bcl-2, Bcl-xL, COX-2 and cyclin D1. In addition, it was even reported to synergize with the antitumor activity of gemcitabine [94]. Several years ago, it has been reported that resveratrol represents a strong activator of SIRT1, a NAD^+^-dependent histone deacetylase with antiapoptotic, anti-inflammatory as well as transcription and cell cycle regulating activities [95]. Activating SIRT1 might be another strategy of resveratrol to inhibit NF-κB- and/or AP-1 signaling pathways [96]. Recent data instead raise doubts about the SIRT1 activating activity of resveratrol. A fluorescent-based *in vitro* assay was used to demonstrate SIRT1 activation by resveratrol [95,97]. However, it has been revealed that this Fluor de Lys-SIRT1 peptide represents an artificial substrate, since resveratrol failed to increase SIRT1 activity in the absence of the fluorophor [98,99]. Using recombinant SIRT1, deacetylation of an acetylated p53-derived peptide or peroxisome proliferator-activated receptor-γ coactivator-1α (PGC-1α) could be observed *in vitro*. Whereas both reactions could be prevented by incubation with a SIRT1 inhibitor, resveratrol did not change the acetylation level of these substrates [99]. Similarly, by using NMR techniques, a more recent study provided evidence that resveratrol indeed interacted with fluorophore-containing peptide substrates but that it was unable to activate SIRT1 in presence of native substrates, such as full-length protein substrates p53 and acetyl-CoA synthetase1 [100]. Consequently, resveratrol seems to be no direct activator of SIRT1. On the other hand, Boily and coworkers demonstrated that the antitumor activity of reveratrol at least partly depends on SIRT1. They have shown that resveratrol strongly prevented the development of induced skin papillomas in mice, while this protective effect was almost eliminated in SIRT1-null mice [101]. Recently, it has been reported that SIRT1 underlies a redox regulation. *S*-nitrosoglutathione (GSNO) for example modified cysteine residues of SIRT1. Instead of affecting its deacetylase activity, such *S*-glutathiolation reactions prevented SIRT1 activation by resveratrol [102]. Moreover, reduced SIRT1 levels were observed in aged and atherosclerotic vessels *in vivo* [103]. Downregulation of SIRT1 was also observed in response to cigarette smoke-induced oxidative stress in bronchial epithelial cells and is rather caused by lipid peroxidation byproducts than directly by ROS [104]. The authors provided evidence that post-translational modifications on cysteine residues induced by reactive aldehydes could lead to inactivation and proteasome-dependent degradation of SIRT1 [104]. Counteracting oxidative stress might therefore mediate the upregulation of SIRT1 mRNA expression, as observed in human umbilical vein endothelial cells (HUVECs) upon resveratrol treatment [103] and explain the positive effects of resveratrol on SIRT1 activation. Resveratrol also suppresses growth of myeloid cells. Findings of Lee *et al.* demonstrated that resveratrol, although inhibiting proliferation of both, promyelocytic leukemia cells (HL-60) and non-malignant B-cell lymphoblastoid (WIL2-NS) cells, by blocking cell cycle progression in G0/G1 phase, induced apoptosis selectively in HL-60 cells. WIL2-NS cells might anticipate cell death due to their ability to repair DNA damage and restore cell cycle progression [46]. In summary, resveratrol seems to have only marginal cytotoxic effects on non-malignant cells. In contrast to HL-60 cells that are killed *via* the CD95-CD95 ligand pathway [105,106], resveratrol has been reported to drive apoptosis also in CD95-signaling-resistant ALL cell lines by activating the intrinsic apoptotic pathway whereas normal peripheral blood mononuclear cells (PBMCs) are not affected [107]. Equally, leukemic lymphoblasts isolated from pediatric patients with ALL undergo apoptosis when treated with resveratrol [45].

### 2.3. Resveratrol Derivatives and Their Potential as Anti-leukemic Agents

**Figure 2:**
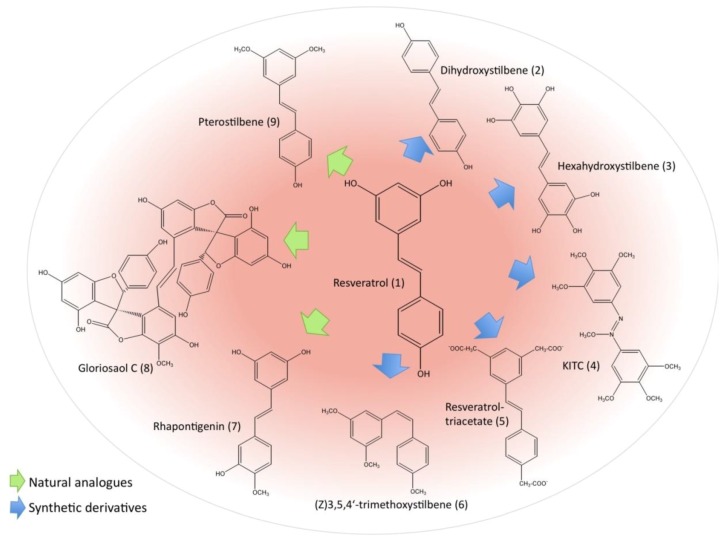
Reseveratrol and derivatives.

As the chemopreventive potential of resveratrol (1) is of remarkable interest, more and more researchers focus their attention also on resveratrol derivatives, which either originate from nature or are chemically synthesized in order to improve its biological activities. The chemical structures of resveratrol and its most potent derivatives are illustrated in Figure 2. Investigations of the structure-activity relationship revealed some structural determinants responsible for biological activity of resveratrol and its derivatives. Thus, it has been reported that the number and position of hydroxyl groups as well as intramolecular hydrogen bonding are essential features [108,109,110]. 

Compared to *trans*-resveratrol, *trans*-stilbene compounds containing 4-hydroxy group, double bonds and *ortho*- or *para*-diphenoxyl functionalities exert significantly higher activity [108]. Similarly, Fang *et al.* found that the position of hydroxyl groups and the oxidation potential of the molecule determine the antioxidant activity of resveratrol analogues. In this respect, especially derivatives with *ortho*-dihydroxyl (3,4-dihydroxy-*trans*-stilbene) and/or *para*-hydroxyl functionalities showed the highest antioxidative effects against 2,2'-azobis(2-amidinopropane) hydrochloride (AAPH)-initated peroxidation of linoleic acid in sodium dodecyl sulfate (SDS) and cetyl trimethylammonium bromide (CTAB) micelles [109]. Their findings revealed that the tested resveratrol analogues act by scavenging lipid peroxyl radicals and were also able to regenerate vitamin E from the α-tocopheroxyl radical back to its active form [109]. In a more recent study, the radical-scavenging activity of nine chemically synthesized resveratrol analogues was analyzed by the reaction kinetics with galvinoxyl (GO^•^) and 2,2-diphenyl-1-picryl-hydrazyl (DPPH^•^) radicals in ethanol and ethyl acetate using UV-vis spectroscopy. The results confirmed previous observations that 3,4-dihydroxy-*trans*-stilbene is the most active resveratrol derivative and the authors suggested that the 4'-hydroxyl group is more favorably oxidised compared to 3-OH or 5-OH [110]. In this respect and in agreement with other studies, radical scavenging activity of resveratrol derivatives can be improved by introduction of methyl, methoxy or hydroxyl groups in the *ortho*- or *para*-position of 4-OH [110]. Previous findings indicated that 4-4’-dihydroxy-*trans*-stilbene (2) as well as 3,4-dihydroxy-*trans*-stilbene possess significantly higher antiapoptotic potential on human promyelocytic leukemia cells (HL-60) than resveratrol itself or other tested derivatives and that the latter compound effectively inhibited ROS-induced DNA damage and enhanced DNA damage in presence of cupric ions [111,112]. The antiproliferative activity of such hydroxystilbenes carrying *ortho*-hydroxyl groups on HL-60 leukemic cells was shown to be more than three-fold higher than those with other groups [113]. This cytotoxic effect could be explained by the observation that *ortho*-hydroxystilbenes are converted into oxidized intermediates (*ortho*-semiquinones), for which is known that they undergo redox-cycling and thus generate further oxygen radicals [113]. Similarly, a 6,600-fold higher antioxidant activity and stronger antileukemic effects compared to resveratrol have been demonstrated for hexahydroxystilbene (3). This analogue acts *via* inhibition of NF-κB activation and induction of cell cycle arrest in HL-60 cells [113,114]. Moreover, this compound also successfully blocked the H_2_O_2_-mediated formation of DNA single-strand breaks in HL-60 cells [113,115]. Another strategy exerted by hexahydroxystilbene is the modulation of the cellular redox balance by decreasing SOD and GSH levels [116]. A polymethoxylated variant (*N*-hydroxy-*N'*-(3,4,5-trimethoxyphenyl)-3,4,5-trimethoxy-benzamidine; KITC (4)) likewise displays significant activity against HL-60 cells [117]. Roberti *et al.* evaluated the antiproliferative and pro-apoptotic potential of 49 synthesized resveratrol derivatives on HL-60 cells, including multidrug-resistant (MDR) HL-60R cells. In general, they found that *cis*-isoforms are more active than the corresponding *trans*-isoforms, with exception of *trans*-resveratrol. Moreover, derivatives with 3'-hydroxy-4'-methoxy groups showed higher activity than 4'-hydroxy-3'-methoxy compounds [118]. Amongst them, especially two compounds exhibited remarkable pro-apoptotic properties at nanomolecular concentrations: the *cis*-3,5-dimethoxy analogues of rhapontigenin (7) and its 3'-amino derivative. This effect was even stronger than the cytotoxicity of classical chemotherapeutic drugs including etoposide and 5-fluorouracil [118]. Both compounds were even able to cause apoptosis in HL-60 cells that display a MDR phenotype. This finding is of particular interest because the MDR reversing agents currently tested in clinical studies have some limitations due to toxic side effects or alterations in pharmacokinetics of cytotoxic agents. These resveratrol analogues might therefore be a promising compounds for the treatment of MDR expressing malignancies [118]. Acetylation of resveratrol, a modification that is likely to improve its absorption, leads to a derivative with similar ability to arrest the cell cycle in S phase. This resveratrol-triacetate (5) further exhibits synergistic effects with 5-fluorouracil in colon cancer cells highlighting its possible role as chemosensitizer [119]. Recently, experimental docking studies of several derivatives revealed that most (*Z*)-isomers fit to the colchicin binding site of tubulin [120]. For (*Z*)-3,5,4'-trimethoxystilbene (6), the most powerful analogue, it is already known to act *via* tubulin depolymerization [121]. Accordingly, this compound and other methylated derivatives cause mitotic arrest instead of S phase arrest induced by resveratrol itself and increase the level of polyploidy. Methylation is considered to stabilize the compounds and increase their bioavailability [120]. Beside these synthetic compounds, two natural polyphenols, structurally related to resveratrol, have been reported as potent anti-leukemic agents: dimethylated pterostilbene (9) from blueberries and gloriosaol C (8) isolated from *Yucca gloriosa* both arrest the cell cycle at G1 phase and induce apoptosis in leukemia and lymphoma cell lines [122,123,124]. The former compound, although scavenging peroxyl-radicals to a similar extent than resveratrol, is of special interest as it causes apoptosis even in MDR-resistant hematologic malignancies [124]. 

### 2.4. Possible Negative Effects of Resveratrol

Many studies provided evidence that resveratrol possesses a huge chemopreventive potential in rodent models as well as in human cancers without causing severe side effects. Findings from Lee *et al.* indicated that resveratrol affected cell cycle progression not only of malignant HL-60 leukemia cells but also of a transformed, non-malignant B-cell lymphoblastoid cell line. However, the observed effect was only marginal since solely leukemic cells ended up in irreversible cell death [46]. It has been shown that resveratrol binds to DNA in the presence of Cu^2+^ ions and consequently induces DNA strand breaks. In complex with Cu^2+^, resveratrol reduces Cu^2+^ to Cu^+^ while the emerging oxidized resveratrol products further enhance the genotoxicity [76]. Such pro-oxidant activity, inducing apoptotic DNA fragmentation in cancerous cells, displays an important antitumor promotion mechanism of chemotherapeutic agents. On the other hand, genotoxic DNA cleavage might affect healthy cells. Lee *et al.*, however, have shown that resveratrol is unable to damage chromosomes in malignant or non-malignant cells [46] and De Salvia *et al.* demonstrated that resveratrol does not lead to primary DNA damage [74]. Only at highest concentrations, chromosomal aberrations are slightly increased. In fact, incubation with resveratrol before H_2_O_2_ application is even able to reduce oxidative DNA damage to control levels [74]. From several studies it is known that resveratrol has remarkable selective growth-inhibitory effects on human tumor cell lines including hematologic malignancies *in vitro*. Observations of Gao *et al.* confirmed the strong antiproliferative effect of resveratrol *in vitro* on 32Dp210 leukemia cells [125]. But, unexpectedly, when mice were inoculated with these leukemia cells and afterwards treated with 8 mg/kg body weight of resveratrol, no antileukemic effect could be detected. Even if resveratrol was administered at much higher doses only a negligible number of mice could be protected from leukemia [125]. This weak *in vivo* response might partly be explained by an improvable bioavailability or fast metabolism of the compound – problems that can be overcome in future by the synthesis of optimized resveratrol derivatives.

## 3. Curcumin

Another natural product belonging to the group of polyphenols is curcumin (10), a yellow pigment derived from rhizomes of turmeric (*Curcuma longa*). This lipid-soluble compound is mainly used in Asian dietary as spice and food-coloring agent. In this respect, it is responsible for the typical yellow color of curry. Curcumin, as part of Ayurvedic medicine, has been subject to a multitude of investigations over the last five decades that demonstrated various health benefits ranging from anti-inflammatory [126,127] and antioxidant [128,129,130] to anticarcinogenic properties [131,132]. Furthermore, antidiabetic [133] and anti-HIV [134] activities were also described. Numerous *in vitro* and *in vivo* studies confirmed its antiproliferative and pro-apoptotic activity in a panel of tumor cells [131,132,135,136,137,138,139,140,141,142,143]. The potent anticancer property of curcumin is attributed to its antioxidant effects that inhibit free radicals from mediating peroxidation of membrane lipids or oxidative DNA damage – both important initiators of cancer development. However, a rising number of recent studies revealed that curcumin exerts its anticancer activity also by acting as a pro-oxidant. Comparison of curcumin and its naturally occurring analogues delivered insight into structure-activity relationships of these compounds. While its high radical scavenging potential is due to a high number of *ortho*-methoxy substitutions as well as a high level of hydrogenation of the heptadiene moiety [144,145], its anti-inflammatory and anticancer activity, in contrast, depends on low hydrogenation and a high level of methoxylation [146]. Owing to its high antioxidant and anti-inflammatory activity as well as its negligible toxic side effects to rodents and humans (when administered at doses up to 10 g/day) [147], growing attention has focused on curcumin as promising anticancer agent [130].

### 3.1. Antioxidant Effects of Curcumin Prevent Initiation of Carcinogenesis

As described before, inhibition of lipid peroxidation is one mechanism by which antioxidants act as chemopreventive agents. It has been reported that curcumin and some of its analogues inhibit free radical-induced LDL peroxidation [148]. Because of its high lipid-solubility, curcumin physically interacts with the cellular membrane where it is converted into a phenoxyl radical in response to quenching of lipid radicals [149]. Since those curcumin analogues bearing no phenolic group are unable to inhibit AAPH- and Cu^2+^-induced LDL oxidation, it has been concluded that this phenolic group rather than the central methylenic group represents the proton donor and is necessary for the activity [149]. Wei and coworkers further demonstrated that the phenolic group is of great importance for the antioxidative effects of curcumin as lipid and protein oxidation of rat liver mitochondria treated with AAPH and Fe^2+^/ascorbate (VC) could be prevented by curcumin and its analogues [150]. Further evidence came from a more recent *in vitro* study in which SDS and CTAB micelles were used to analyze the antioxidative effects of curcumin against the free-radical-induced peroxidation in linoleic acid. The results verified previous findings that curcumin and its analogues act *via* proton abstraction from its phenolic group [151]. Recent observations indicated that curcumin at a concentration of 20 mM prevented lipid peroxidation of linoleic acid emulsion by 97.3%. By performing detailed *in vitro* antioxidant assays the authors also demonstrated effective radical scavenging properties of curcumin including the 1,1-diphenyl-2-picryl-hydrazyl (DPPH^•^) radical, 2,2'-azino-bis(3-ethylbenzthiazoline-6-sulphonic acid (ABTS^•+^) and *N,N*-dimethyl-*p*-phenylenediamine dihydrochloride (DMPD^•+^) radical cations, O_2_^•−^ and H_2_O_2_ [152]. Metal ions play an important role as inducers of ROS formation and due to its huge reactivity, iron is the major pro-oxidant among transition metals involved in lipid damage [13]. Ak and Gülçin could show that curcumin has a high binding affinity for ferrous ions (Fe^2+^) and they conclude that this chelating ability might be the main mechanism by which curcumin inhibits lipid peroxidation [152]. 

The antioxidative properties of curcumin act not only against lipid peroxidation but also prevent DNA damage. Thus, a field trial with West Bengalian patients indicated that three months of curcumin administration prevented ROS generation as well as subsequent DNA damage and lipid peroxidation in people exposed to arsenic contamination of groundwater compared to untreated persons [153]. Further studies revealed that the antioxidant properties of curcumin positively influenced antioxidant and phase II metabolizing enzyme activity in mice and moreover diminished iron-induced oxidative damage of lipids and DNA *in vitro* and in mice treated with ferric nitrilotriacetate (Fe-NTA) [154,155]. In detail, curcumin was able to prevent Fe-NTA-induced lipid peroxidation, DNA damage and protein carbonylation in the kidney of these mice [130].

### 3.2. Curcumin Exerts its Anticancer Properties also as Pro-oxidant

Interestingly, although curcumin is considered as antioxidant there is a growing number of evidence that curcumin can act as pro-oxidant under certain conditions, exerting its anticancer activity by inducing ROS generation [156,157,158]. Chen *et al.* recently investigated the antioxidant and anticancer properties of curcumin on HL-60 human leukemia cells by measuring cell proliferation, viability and ROS generation. Interestingly, they found that the anticarcinogenic mechanisms of curcumin differ depending on its concentration. Whereas low concentrations of curcumin (<20 μM) decrease ROS production, higher concentrations have the opposite effect and favor ROS generation [157]. The authors furthermore investigated the influence of three water-soluble antioxidant compounds on antioxidant and anticarcinogenic activity of curcumin and found that ascorbic acid, *N*-acetyl cysteine as well as GSH augmented both activities of low concentrations of curcumin [157]. Hence, a combination of lower levels of curcumin with water-soluble antioxidants might represent an adequate strategy to improve its anticancer property without increasing harmful ROS accumulation. Recently, it has been reported that curcumin exerts cytotoxic activity towards CCRF-CEM human T-cell leukemia but hardly affects normal cells. By using gel electrophoresis analyses Kong *et al*. found that oxidant curcumin-Cu(II) ions induced DNA damage in plasmid DNA whereas curcumin alone failed [159]. Free radicals can induce epigenetic effects at the DNA level by loosening the chromatin structure and consequently enhancing the accessibility for transcription factors that finally regulate expression of genes involved in proliferation [13,160]. Thus, oxidative DNA damage plays an important role in the development of carcinogenesis. The chromatin structure is known to be opened due to histone acetylation, which stimulates gene transcription of silenced genes and can be modulated byROS [160,161]. A few years ago, Kang *et al.* presented a new cellular target of curcumin. They proved that ROS, generated upon treatment of Hep3B cells with higher curcumin concentrations, significantly reduced histone acetylation by inhibiting histone acetyl transferase [160].

Thioredoxin reductase (TR) was discovered to be another main target molecule of curcumin. The ability of TR1 to catalyze the reduction of the disulfide at the active site of TRX was irreversibly inhibited in rat in the presence of curcumin, simultaneously affecting redox functions of TRX. Alkylation of both cysteine and selenium-cysteine residues at the catalytically active site resulted in a modified curcumin-TR enzyme. This enzyme had lost TRX reducing activity but gained a strongly increased NADPH oxidase activity, leading to ROS generation. In essence, curcumin was able to convert TR into a pro-oxidant [162]. Oxidized TRX serves as electron donor for scavenging enzymes, such as thioredoxin peroxidases and methionine sulfoxide reductases. Inhibition of TR function will therefore suppress the cellular antioxidant defense. As a consequence, raised ROS levels induce direct damage to DNA and impair the NF-κB mediated survival mechanism of cancer cells [162].

Syng-ai *et al.* demonstrated that curcumin induces ROS generation in MCF-7, MDAMB and Hep2 cell lines followed by apoptosis, while normal rat hepatocytes were not affected. They found that GSH levels were increased in response to curcumin treatment and that GSH depletion by buthionine sulfoximine (BSO), an inhibitor of γ-glutamylcysteinyl synthetase, enhanced curcumin sensitivity and cell death rate [163]. In K562 leukemic cells Awashi *et al.* detected modulation of γ-glutamylcysteinyl synthetase activity by curcumin and the presence of glutathiolated curcumin products that caused GSH efflux and subsequent increase of GSH synthesis [164]. Thus, endogenous GSH interferes with curcumin and counteracts ROS production. From these observations the authors concluded that ROS is the main trigger of apoptosis [163]. Earlier studies observed GSH efflux simultaneous with the onset of apoptosis and demonstrated that this redox imbalance by GSH depletion is both essential and sufficient for activation of cytochrome c release as key event of a damage-induced apoptoticpathway [165,166]. Armstrong *et al*. used BSO to investigate the chronological order and importance of mitochondrial events and apoptotic signals. They identified GSH as key regulator of apoptosis in PW cells, since the loss of mitochondrial GSH early activated apoptosis. NF-κB activation as well as cytochrome c release from mitochondria of PW cells occurred in response to GSH depletion but before increased levels of ROS were detected [167]. Similarly, Franco *et al*. revealed that induction of apoptosis by GSH depletion is independent of ROS [168]. Various groups suggested that curcumin influences GSH levels *via* the antiapoptotic protein Bcl-2, since a positive correlation was observed between Bcl-2 and GSH [169,170]. Here, following ROS accumulation, downregulation of antiapoptotic Bcl-2 was observed in MCF-7 and MDAMB cells, leading to sensitization of the cells to apoptosis [163]. Bcl-2 expression is likewise known to depend on NF-κB activation, which for its part is also inhibited by curcumin [171]. On the other hand, Piwocka *et al.* provided evidence that curcumin-induced increase of GSH levels is responsible for the induction of a nontypical apoptotic death pathway in lymphoid Jurkat cells. These cells showed internucleosomal DNA fragmentation whereas neither caspase-3 nor mitochondria are involved. Bcl-2 expression levels did not decrease, even after GSH depletion, indicating that GSH acts downstream of Bcl-2 and upstream of mitochondrial events [172]. It is further worth mentioning that some transcription factors, including NF-κB, are redox-sensitive due to their cysteine-thiols. Therefore, changes in cellular redox state caused by curcumin with increased GSH efflux will influence the activation of such redox-sensitive transcription factors [173]. Subsequent expression of their gene products consequently affects cellular signaling pathways. This mode of action might finally contribute to the antitumor promotion activity of curcumin [174]. 

### 3.3. Regulation of Cell Cycle, Proliferation and Apoptosis by Curcumin Affects Cancer Promotion

Inhibition of cell proliferation and induction of apoptosis represent the two strategies of chemotherapeutic agents to prevent tumor promotion. Curcumin possesses manifold ways to impair this stage of cancer development as well as invasion, metastasis and angiogenesis of tumors by disturbing different signaling pathways. Recently, Ravindran *et al.* gave an excellent overview of the different ways utilized by curcumin for killing tumor cells [175]. Among more than 30 known cellular targets of curcumin are transcription factors like NF-κB, growth factors, cytokines, enzymes and genes with a role in cell growth and programmed cell death. Syng-ai *et al.* found a loss of c-*myc* in MCF-7, MDAMB and Hep2 cancer cell lines that might be a hint for cell cycle arrest at G1/S transition as preliminary event before the cells undergo apoptosis [163,176]. Additionally, treatment with curcumin or its derivatives bis-demethoxycurcumin (BDMC) and di-acetylcurcumin (DAC) lead to cell cycle arrest in G0/G1 and/or G2/M phase. The latter is caused by disturbance of microtubule dynamics that finally prevents chromosome segregation and results in cell cycle arrest at early anaphase [175,177]. Sun and coworkers analyzed the effect of curcumin on human B cell non-Hodgkin’s lymphoma. Their findings indicated that curcumin selectively inhibited proliferation of human Burkitt’s lymphoma Raji cells by arresting cell cycle at both G0/G1 and G2/M phase and subsequent apoptosis. In contrast, proliferation of normal peripheral blood mononuclear cells (PBMCs) was not inhibited [178]. 

Recently, it has been demonstrated that curcumin exerts likewise pro-apoptotic activity on both leukemia cell lines K562, a Philadelphia-positive CML, and Jurkat T-cell leukemia as well as in follicular lymphoma cell lines [179,180]. In ALL cells, curcumin causes programmed cell death by inhibiting the PI3’-kinase/AKT pathway [181]. Findings from Harikumar *et al*. have further shown that curcumin acts by decreasing the expression of antiapoptotic Bcl-2 and proto-oncogene Raf-1 and concomitantly activates p53 expression in BALB/c mice suffering from retrovirus-induced erythroleukemia [47]. Curcumin treatment improved significantly the survival time of these mice. Furthermore, incidence of anemic conditions as well as leukemic cell infiltrations in the spleen were decreased indicating suppression of cancer progression [47]. In the same way curcumin administration enhanced the survival of mice with acute lymphoblastic leukemia. Cultured BCR-ABL B-cell ALL cells were killed by apoptosis in response to curcumin and again p53 levels were elevated whereas NF-κB was decreased [182]. The intrinsic apoptotic pathway was implicated in curcumin-induced cell death of HL-60 leukemia cells with caspase-8, BID cleavage, subsequent caspase-3 activation and cytochrome *c* release [183].

As various signaling pathways are constitutively activated in most malignant phenotypes, our laboratory performed a Kinexus phosphosite screen on nuclear extracts derived from human chronic myelogenous leukemia K562 cells before and after 48h treatment with curcumin in order to investigate its effect on cellular phosphoproteins. We found that curcumin induced protein phosphorylation of six and dephosphorylation of seven phosphoproteins, each of them playing an important role in signal transduction [184]. Of special interest is the regulation of signal transducers and transcriptional activators (STATs), proteins with multiple roles in differentiation, cell growth and apoptosis as well as inflammation and immune response that are constitutively activated in chronic myeloid leukemia but also in other cancers. When we checked for nuclear expression of different STATs in K562 leukemia cells, we found STAT3, -5a and -5b expression significantly decreased in the nucleus of curcumin treated cells with a maximal reduction after 48 hours. However, significant changes in their phosphorylation levels could not be detected [185]. Instead, simultaneously with reduced nuclear expression, the level of truncated isoforms of STAT5 resident in the cytoplasm was elevated. These isoforms serve as negative regulators of the native STAT5 because they still exert their DNA-binding ability and compete with native forms for its DNA-binding sites. Curcumin therefore might represent a powerful tool to fight STAT5 overexpressing cancers [185]. In accordance with these findings, Rajasingh *et al.* reported that curcumin exerted its anti-proliferative and proapoptotic functions on T cell leukemia similarly by inhibiting the JAK/STAT pathway [186]. Additionally, based on inhibition of kinase Jak 1 and its effect on STAT3 activity, growth arrest and subsequent apoptosis were induced in primary effusion leukemia upon curcumin treatment [187].

Beside STATs, transcription factors NF-κB and AP-1 are well known targets inhibited bycurcumin [188]. Accordingly, Ghosh *et al.* demonstrated that STAT3, AKT and NK-κB were inhibited in curcumin-induced apoptosis in CLL B cells together with antiapoptotic proteins Mcl-1 and XIAP [189]. Curcumin likewise mediated caspase-dependent apoptosis in cutaneous T-cell lymphoma (CTCL) by downregulating STAT3 and NF-κB signaling [190]. The inhibiting activity of curcumin on NF-κB signal transduction is of special importance as we previously observed that this pathway is involved in TNF-α-mediated induction of γ-glutamyltransferase (GGT), an enzyme whose overexpression is implicated in cancer drug resistance and inflammatory leukotriene synthesis [191]. We performed a real-time PCR array study to analyze the effect of curcumin on transcription of NF-κB controlled genes in K562 cells. We found that mRNA expression of 39 of 84 genes involved in different NF-κB signaling pathways was modulated by curcumin [192]. Some genes could be identified for the first time as cellular targets of curcumin, amongst them *AGT*, *CSF3*, *TICAM2*, *TNFRSF7*, which were activated, whereas *CD40*, a member of TNF receptor superfamily, represented the most inhibited gene. By genome-wide microarray analysis performed under the same conditions we demonstrated that especially cell cycle genes and genes from the JAK/STAT signaling pathway were downregulated whereas heat shock proteins are among the 10 most upregulated genes [193]. Consequently we investigated the induction of heat shock response by curcumin in K562 cells in detail. We demonstrated that the heat shock transcription factor (HSF)-1 was translocated to the nucleus and activated *hsp70* promoter through binding to a heat shock regulatory element (HSE) in response to curcumin treatment [194]. The subsequent upregulation of Hsp70 is associated with inhibition of NF-κB activation [195,196,197] and plays a key role in the anti-inflammatory activity of this compound. Previous work of our laboratory has demonstrated that curcumin blocks DNA interaction of transcription factors AP-1 and NK-κB with the glutathione *S*-transferase (GSTP1-1) promoter region in K562 leukemia cells and consequently prevents transcription of *GSTP1-1* gene [198]. This effect seems to depend on the serum level of the culture medium as 10% FCS in the medium induced an opposite effect compared to medium containing 0.1% FCS and an increase of GSTP1-1 mRNA expression could be observed. We suggested that binding of curcumin to biological molecules could explain this observation [199]. Such interactions could interfere with cellular uptake or degradation of curcumin as reported by Ravindranath and Chandrasekhare in rats [200]. Increased levels of this enzyme are present in chemotherapy-resistant cancer cell lines and it is known that GSTP1-1 functions in the export of xenobiotic drugs after their conjugation to GSH [201]. As a result of curcumin treatment, K562 cells undergo caspase-dependent apoptosis with activation of both initiator caspases-8 and -9 [198].

Like resveratrol curcumin also exerts anti-inflammatory activity that is associated with development of cancers. It has been reported in the literature that molecular targets of curcumin include not only transcription factors but moreover ROS-generating enzymes like cyclooxygenase-2, lipoxygenase (LOX) as well as iNOS [147,174,202,203].

### 3.4. Overcoming Complications/Chemotherapy Resistance of Leukemia by Curcumin Treatment

Due to interactions with their microenvironment, especially with integrin-binding ligands expressed by marrow stromal cells, CLL B cells become resistant against the most common chemotherapeutic treatments [204,205,206]. This effect, also kown as stromal protection, prevents apoptosis occurring either spontaneously or induced by drugs. Results of Ghosh *et al.* indicated that both soluble factors and direct cell contact in a coculture of CLL B cells with stromal cells enhance activation of STAT3 and levels of some antiapoptotic proteins. Consequently, these cells are protected from apoptosis. However, evidence has been provided that a higher dose of curcumin is efficient in blocking this stromal protection [189]. The authors expanded their study on a second polyphenolic compound extracted from green tea: epigallocatechin-3 gallate (EGCG), which is also known to induce apoptosis in CLL B cells. A combinational treatment with curcumin and EGCG resulted in more than additive effects if both compounds were administered sequentially [207]. In this respect, better results could be achieved when cells were pretreated with EGCG. This sequential treatment not only potentiated apoptosis but also allowed curcumin to overcome stromal protection, even at lower concentrations [189]. This synergistic effect was confirmed by Somers-Edgar’s group in breast cancer cells. Treatment with a combination of curcumin and EGCG significantly enhanced the percentage of cells arrested in G2/M. This finding was verified *in vivo*, where simultaneous administration of both compounds reduced tumor volume in athymic nude female mice by 50% [208]. 

One problem of standard chemotherapy is the development of resistance. In this respect, curcumin was reported to act as chemosensitizer since combinational treatment efficiently enhanced both the pro-apoptotic and NF-κB inhibitory potential of capecitabine in human colorectal cancer. Combinational administration in nude mice even drastically decreased tumor volume and metastasis [141]. The efficiency of radiation therapy is likewise restricted by resistance mechanisms, which underlies stimulation of NF-κB activity. Because of its ability to suppress this pathway by preventing phosphorylation and subsequent degradation of IκB-α, curcumin modulates the radiosensitivity of colorectal cancer cells [209].

Another known complication of classical chemotherapy is the late appearance of secondary cancers. Siddique *et al.* recently reported that curcumin protects human blood lymphocytes from genotoxic effects caused by mitomycin C, an antineoplastic agent, thereby improving the outcome of the cancer therapy when used in combination with traditional chemotherapeutic drugs [210]. Another severe problem of leukemia is that patients are often immunosuppressive and are thus more susceptible to infections. In addition to all these pathways that curcumin interferes with and on which its chemopreventive activity is based on, a very recent report suggested that curcumin has great potential to act as an effective modifier in therapy of leukemia and as an immunopotentiator due to its differentiation-stimulating ability [211]. The authors provided evidence that curcumin significantly activated the O_2_^−^-generating activity in leukocytes during retinoic acid-induced differentiation of U937 leukemia cells by accumulating two cytoplasmic components, p47-phox and p67-phox. This O_2_^−^-generating system, which is necessary for activation of phagocytosis, is typically absent in human monoblastic leukemia cells. As a consequence, leukemia patients might be protected from life-threatening infectious diseases [211].

### 3.5. Evaluation of Natural and Synthetic Curcumin Derivatives and Other Strategies to Improve Bioavailability of Curcumin

**Figure 3:**
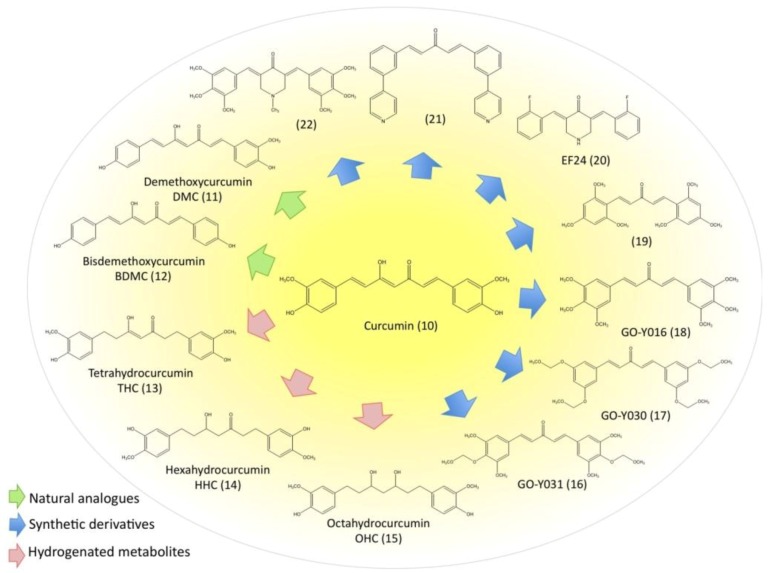
Curcumin and derivatives.

Despite its huge antioxidant and anticancer potential, the use of curcumin as chemopreventive and chemotherapeutic agent is limited mainly by its weak bioavailability due to poor absorption and rapid metabolism as well as its low water solubility. Currently, several strategies are being tested to overcome these limitations. During the last years, not only natural analogues from turmeric have been tested for their anti-carcinogenic activity compared with that of curcumin. Moreover, a huge amount of structurally modified curcumin derivatives has been chemically synthesized and evaluated in order to create a curcumin-derived molecule with better *in vivo* bioavailability and improved selectivity. Figure 3 provides an overview of the chemical structures of the curcuminoids and a selection of the most important curcumin derivatives. Beside chemical modifications of the compound, another tactic is the development of formulations that should enhance the absorption of curcumin by lowering its hydrophobicity and increasing membrane permeability. In this respect, the most promising applications worth mentioning include nanoparticles [212], liposomes [213], micelles [214] and phospholipid complexes [215]. Anand *et al*. for example designed poly (lactide-co-glycolide) (PLGA) nanoparticles with encapsulated curcumin. These nanoparticles confer not only better bioavailability and longer halflife to curcumin but also accelerate its uptake and enhance its antiproliferative and proapoptotic potential in leukemia cells [216]. Recently, Yadav *et al*. successfully improved cellular uptake of curcumin by novel cyclodextrin-complexes [217]. In addition, simultaneous application of special adjuvants can prevent the rapid metabolism of curcumin and thereby increases its biological activity [218].

Rhizomes of turmeric contain a mixture of three compounds referred as curcuminoids: curcumin (10) being the most abundant compound (77%), followed by demethoxycurcumin (11) (DMC, 17%) and bisdemethoxycurcumin (12) (BDMC, 3%) [144]. These compounds, together with the curcumin metabolite tetrahydrocurcumin (13) (THC) have been the subject of many structure-activity-relationship studies. Somparn *et al.* recently demonstrated the key role of methoxy groups on the phenyl ring for the antioxidant effects of curcuminoids. Decreasing activities have been observed in the order curcumin>DMC>BDMC since curcumin showed the strongest effects on scavenging DPPH radicals, preventing lipid peroxidation and protein oxidation [145]. Curcumin was likewise more effective in suppressing TNF-induced activation of NF-κB than DMC, BDMC and THC, whereas all curcuminoids inhibited growth of various cancer cell lines including T-cell leukemia (Jurkat), histiocytic leukemia (U937) and CML (KBM-5) to a similar extent. Only THC exhibited less activity in this respect. Further, no significant difference was found in GSH production [144]. The lacking inhibitory effect of THC emphasized the importance of double bonds in the modulation of NF-κB activation. Further findings indicated that BDMC was most effective in ROS generation [144,219] and the authors therefore concluded that the GSH status but not ROS level is related to antiproliferative effects of curcuminoids [144]. It has further been reported that BDMC possesses higher antitumor, antipromotion and anticarcinogenic potential than curcumin or DMC [220,221] whereas another group found that curcumin and not BDMC was the most effective pro-oxidant causing ROS-induced DNA cleavage [222]. Anuchapreeda *et al.* focussed their research on the effect of curcuminoids on leukemic cells, especially on gene expression of oncogene *Wilm’s tumor1* (*WT1*) [223], as the corresponding protein is known to be overexpressed in immature leukemia cells and represents a crucial player in leukemogenesis [224,225]. They provided evidence that among all curcuminoids, curcumin most efficiently decreased WT1 mRNA expression and protein levels in K562 and Molt4 cells [223]. In human hepatocytes, curcumin is metabolized to different hydrogenated derivatives including THC, hexa- and octahydrocurcumin (14,15) (HHC, OHC) [226]. In contrast to the natural curcuminoids, the antioxidant properties of THC, HHC and OHC were significantly higher than that of curcumin stressing the fact that hydrogenation of the heptadiene moiety of curcumin is responsible for an improved antioxidant function [145,146] while this modification simultaneously decreases its antitumor and anti-inflammatory properties [220]. These results are in line with previous findings that THC was more potent to inhibit lipid peroxidation than curcumin [146]. It might therefore be concluded that these metabolites are mainly accountable for the *in vivo* radical scavenging activity of curcumin. 

Beside these naturally occurring compounds, a multitude of chemical modifications have been introduced into the curcumin molecule in order to find a selective anticancer molecule with better pharmacokinetics. In summary, the most important outcomes from these studies are the following: C-3' and C-4' atoms of both phenyl groups should be substituted with 3'-4'-dimethoxy or better 3'-methoxy-4'-hydroxy units to reach a high antioxidant [149] and antiproliferative [227] activity. Since 3,5-bis(4-hydroxy-3-methoxy-5-methylcinnamyl)-*N*-ethylpiperidone exhibits stronger radical scavenging activity on several leukemic cells than curcumin, Youssef *et al.* assumed that the stabilization of the generated phenoxy radical is responsible for the high antioxidative potential and that such stabilization can be achieved by either a *para*-hydroxy phenyl moiety or *ortho*-substitutions [228]. Fuchs *et al.* reached stabilization and subsequent increase of antiproliferative ability of derivatives carrying a heptadienone moiety through conversion of their 4-OH group into methoxy, acetate or sulfamate groups [227]. Introduction of *ortho*-alkoxy groups was linked to substantial increases of ROS scavenging and anticancer properties [229,230,231]. The minimal structural prerequisites of an effective curcumin derivative involve two hydroxyphenyl units connected through an unsaturated linker region, while additional oxygroups can further improve the antioxidant potential [220,232]. From a biological analysis of around 50 curcumin analogues, tested on a panel of cancer cell lines, it turned out that the 18 most active compounds contained a 1,5-diarylpentadienon skeleton [229]. Amongst them, the three most promising compounds GO-Y016 (18), GO-Y030 (17) and GO-Y031 (16), which exceeded the antiproliferative effect of curcumin by far, inhibited growth of most cancer cells lines even more successfully than classical chemotherapeutic agents like 5-fluorouracil. On the other hand, they did not suppress primary hepatocytes nor exert any severe side effects when administered to mice [229]. Fuchs *et al.* found likewise a pentadienone analogue to be the most potent one with the highest selectivity against prostate and breast cancer cell lines (19) [227]. Concerning the anticancer activity, bis-benzylidenepiperidone, pyrone, cyclohexanone derivatives and especially 2,6-bis(2-fluorobenzylidene) piperidone (EF24) (20) have been reported to induce cell cycle arrest and apoptosis of cancer cells much more efficiently than curcumin [233]. EF24 was even proven to possess satisfactory oral bioavailability and acceptable pharmacokinetics in mice [234]. Moreover, Lin *et al.* identified bis(3-pyridyl)-1,4-pentadien-3-one (21) out of 72 curcumin analogues as the most potent inhibitor of TNF-induced NF-κB activation [232,235]. We tested recently heterocyclic cyclohexanone analogues of curcumin synthesized by the Larsen and Rosengren groups for their NF-κB inhibiting potential in K562 cells. Amongst them, 3,5-bis(3,4,5-trimethoxybenzylidene)-1-methylpiperidin-4-one (22) displayed the strongest effect with an EC_50_ value of less than 7.5 μM. This compound and other cyclohexanone derivatives moreover efficiently induced apoptosis in estrogen receptor-negative breast cancer cells [236,237]. A very important finding and a promising strategy to improve the bioavailability of curcumin is that water-solubility of curcumin largely increases upon glycosylation of its aromatic ring structure [238]. 

### 3.6. Disadvantages and Possible Negative Effects of Curcumin

Despite its huge proved potential as anti-inflammatory, chemopreventive and anti-carcinogenic agent, there is currently one major disadvantage for the use of curcumin in cancer therapy: its low bioavailability and fast metabolism. Everett *et al.* assessed the potential of curcumin in the treatment of B-CLL, also in combination with common chemotherapeutics. They found that curcumin efficiently induces apoptosis in B-CLL cells within 24-48 hours and even enhances the apoptotic effects of vincristine and other agents at a concentration of 1 μM [239]. It is hardly possible to achieve this concentration by using oral administration even at large curcumin doses. Intravenous infusions might be required. The clinical treatment of patients suffering from advanced pancreatic carcinoma with curcumin in combination with gemcitabine resulted in a response in only 10% of the patients [240]. However, this bioavailability problem is not unsolvable and many efforts have been undertaken to improve the solubility of curcumin and its absorption by either drug optimization studies, curcumin formulations or by simultaneous administration of adjuvants like the black pepper ingredient piperine, which inhibits curcumin glucoronidation and thus boosts its bioavailability by 2000% [241]. 

Most preclinical and clinical studies reported beneficial effects of curcumin against tumors in animals and human beings [136] and clinical trials clearly demonstrated that curcumin is well tolerated and safe at doses of 12 g/day [242]. Nevertheless, one study found that curcumin causes promotion of doxycycline-induced lung tumors in mice with increased tumor multiplicity and oxidative damage in lung tissue [243]. This might rather be an organ-specific effect of curcumin and the authors subsequently recommended that (ex-) smokers should be excluded from chemopreventive trials in order to prevent further damage of lung tissue by curcumin-induced ROS generation [243]. A comprehensive toxicity prediction study of turmeric-derived compounds revealed that curcumin, DMC and BDMC are non-mutagenic but can be carcinogenic in rodents and might possibly exhibit hepatotoxicity in a dose-dependent manner when taken for prolonged periods of time [244]. Based on a toxicology study of curcumin from 2003, the Joint FAO/WHO Expert Committee on Food Additives (JECFA) defined an Acceptable Daily Intake (ADI) for curcumin of 0-3 mg/kg bodyweight [244].

## 4. Conclusions

**Figure 4:**
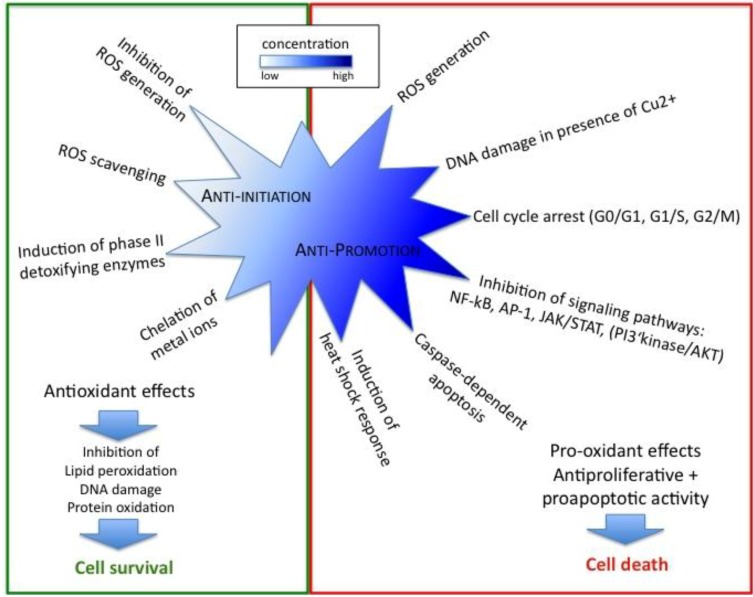
Overview of the chemopreventive and –therapeutic effects of resveratrol and curcumin.

Resveratrol and curcumin represent two particularly important polyphenolic antioxidants with respect to prevention and treatment of human cancers, including hematological malignancies. The incidence of leukemia, with approximately 48,000 new cases per year in European men, is quite high and even if there is currently a panel of various chemotherapeutic agents in use or in clinical trials, they fail to prevent the 32,000 deaths, which occur each year in Europe due to this disease in men. Because of adverse side effects of these drugs on normal tissue and taking into account that a therapy, which might successfully inhibit cancer promotion in the beginning, often ends up in a relapsing stage with enhanced mortality, it becomes clear that we are far away from healing leukemia and that the search for alternative treatments is indispensable. It has been reported that oxidative stress and a decreased level of cellular antioxidant defense are linked to the etiology of leukemia. Moreover, leukemic relapse is likely to result from accumulated oxidative damage following chemotherapeutic treatment. Thus, administration of antioxidants represents a promising strategy to overcome hematologic malignancies even before they start to develop. In this review we clearly demonstrated the remarkable potential of both natural antioxidants resveratrol and curcumin in chemoprevention and chemotherapy of leukemia. Even if in both cases the clinical use is currently limited by poor bioavailability, some new derivatives have already been synthesized that promise a good response in patients. As both substances and their derivatives mediate their multiple effects at quite low doses, one important strategy might be a synergistic combination with classical chemotherapy or other natural antioxidants, which has been already demonstrated for human leukemia cells *in vitro* [189,245,246]. Especially curcumin is of interest for the treatment of leukemia because of its ability to block stromal protection and thus prevent resistance to chemotherapy. On the other hand, it also acts as immunopotentiator and further protects lymphocytes from genotoxic effects, which are known inducers of secondary cancers. Nevertheless, one should always bear in mind that effects of antioxidants differ depending on the stage of carcinogenesis and the concentrations used. Lower concentrations of antioxidants strongly prevent initiation of carcinogenesis, but administration during cancer progression rather prevents apoptosis of tumor cells. In contrast, higher concentrations of these polyphenols often exhibit pro-oxidant and thus remarkable pro-apoptotic anticancer activity. Figure 4 summarizes the anti- and pro-oxidant effects of both compounds and their role for chemoprevention and chemotherapy.
